# Tuberculosis Disease Among Adults Aged 65 Years and Older: Alameda County, California, 2016–2019

**DOI:** 10.1093/ofid/ofac575

**Published:** 2022-10-31

**Authors:** Iris L Wu, Jennie Chen, Rita Shiau, Amit S Chitnis, Devan Jaganath

**Affiliations:** School of Public Health, University of California, Berkeley, California, USA; School of Medicine, Virginia Commonwealth University, Richmond, Virginia, USA; Tuberculosis Section, Division of Communicable Disease Control and Prevention, Alameda County Public Health Department, San Leandro, California, USA; Tuberculosis Section, Division of Communicable Disease Control and Prevention, Alameda County Public Health Department, San Leandro, California, USA; Tuberculosis Section, Division of Communicable Disease Control and Prevention, Alameda County Public Health Department, San Leandro, California, USA; Tuberculosis Section, Division of Communicable Disease Control and Prevention, Alameda County Public Health Department, San Leandro, California, USA; Division of Pediatric Infectious Diseases, University of California, San Francisco, California, USA; Center for Tuberculosis, University of California, San Francisco, California, USA

**Keywords:** aged, epidemiology, public health, surveillance, tuberculosis

## Abstract

**Background:**

Older adults aged ≥65 years old represent an increasing proportion of tuberculosis (TB) cases in the United States, but limited evidence exists on the characteristics and treatment outcomes that differentiate them from younger adults.

**Methods:**

We evaluated Alameda County TB surveillance data from 2016 to 2019 and abstracted public health charts for older adult TB cases. Clinical presentation and treatment outcomes were compared in older and younger adults (15–64 years), and multivariable logistic regression was conducted to assess risk factors for TB treatment noncompletion among older adults.

**Results:**

Of 517 TB cases, 172 (33.2%) were older adults and 101 were ≥75 years old. Compared to younger adults, older TB cases were more likely to be non-US-born, and have diabetes. For diagnosis, older adults were more likely to have negative interferon-gamma release assays (24.6% vs 16.0%; *P* = .01) and were less likely to have cavitary disease (18.6% vs 26.7%; *P* < .001). One third of older adults experienced an adverse event; older adults were less likely to complete TB treatment (77.7% vs 88.4%; *P* = .002) and were more likely to die during TB treatment (16.3% vs 2.9%; *P* < .01), especially among those ≥75 years old, who had a mortality rate of 22.9%. In multivariable analysis, dementia was significantly associated with treatment noncompletion (adjusted odds ratio, 5.05; 95% confidence interval, 1.33–20.32; *P* = .02).

**Conclusions:**

Diabetes, negative diagnostic tests, and poor treatment outcomes were more prevalent in older adult TB cases. A greater understanding of their TB presentation and comorbidities will inform interventions to improve outcomes among older adults.

## Teaser

TB cases aged ≥65 years old were more likely to have noncavitary disease, negative diagnostic tests, treatment noncompletion, and death. Cases with dementia had 5-times higher odds of noncompletion. Further interventions are needed to improve treatment completion and outcomes.

Adults aged 65 years and older (older adults) represent an increasing proportion of tuberculosis (TB) cases in the United States. Since 2017, older adults have comprised over one quarter of all US TB cases while representing only 16% of the population [1]. The demographic shift in TB cases toward older individuals has created new challenges to TB diagnosis and management [2]. Tuberculosis signs and symptoms such as fever, cough, lymphadenopathy, and weight loss overlap with other diseases in this age group including chronic lung diseases and malignancy. Older adults are also less likely than younger adults (15–64 years old) to be identified in contact investigation studies because fewer than 5% of cases are due to recent transmission [3, 4]. Furthermore, older adults are more likely to have comorbidities than younger adults and take multiple medications that may interact with anti-TB treatment [5]. With these challenges, the few studies on TB among older adults suggest that they have worse treatment outcomes than younger adults [6, 7]. Limitations of these studies are that they did not have greater detail on comorbidities, treatment characteristics, and outcomes because they relied on public health surveillance data.

In a large county in Northern California, we utilized both surveillance and public health case management chart data to investigate the clinical, diagnostic, and treatment outcomes of older adults to guide interventions to improve TB care for older adults.

## METHODS

### Setting

Alameda County has a population of 1.7 million persons who reside in rural, suburban, and urban settings, including the city of Oakland. Approximately 37% of the county residents are Asian, and 22% of the population are Hispanic or Latino. One third of the county is non-US born, and 13.9% are aged 65 years and older [8]. During 2016–2019, TB incidence rates in Alameda County varied from 7.4 to 10 TB cases per 100 000 population [9].

### Data Sources

All confirmed adult TB cases reported to Alameda County Public Health Department (ACPHD) TB program during January 1, 2016–December 31, 2019 were included in our analysis. Tuberculosis case data are recorded using the Report of Verified Case of Tuberculosis (RVCT) form, which collects information on (1) demographic and clinical characteristics and (2) treatment outcomes. Local public health departments subsequently report RVCT data to the California Department of Public Health (CPDH) for statewide surveillance purposes. Alameda County RVCT data were obtained from the California Reportable Disease Information Exchange data system for all TB cases aged 15 years and older during January 1, 2016–December 31, 2019. These data were used to summarize demographics, diagnostic and clinical characteristics, and outcomes for all adult cases.

The ACPHD public health case management charts for all older adult TB cases aged 65 years and older within this timeframe were reviewed. Insurance information, clinical characteristics, and treatment outcomes not already present in the RVCT form were abstracted. Presence of specific comorbidities was noted, as well as the number and type of non-TB medications being taken by each patient. Adverse clinical events were characterized by symptom type, duration, and medication regimen before and after each event.

### Definitions

All TB cases were defined using US TB surveillance system definitions [10], in which all confirmed TB cases occurring among persons aged 15 years and older were designated as adult TB cases. A tuberculin skin test (TST) positive result was defined in accordance with Centers for Disease Control and Prevention guidelines: ≥5 mm for immunosuppressed individuals, recent contacts of infectious TB cases, and individuals with prior TB disease; ≥10 mm for individuals with other known risk factors for TB; and ≥5 mm for individuals with no known risk factors for TB. Culture-positive for TB was defined as isolation of *Mycobacterium tuberculosis* complex from cultures of a clinical specimen. In contrast, culture-negative TB was defined as failure to isolate *M tuberculosis* complex from a clinical specimen. Comorbid diseases and severity were collected from public health chart review and used to calculate the Charlson comorbidity index (CCI) [11], which served as a proxy for clinical complexity. The CCI formula excluding age was used to preserve age as an independent variable.

Because surveillance data and medical records available to ACPHD have limited information that allow for an assessment of potential causes of deaths, we were unable to determine whether TB, comorbidities, or other factors contributed to deaths. However, a previous study in the United States that combined surveillance data with a detailed review of medical charts for 1300 deaths among TB patients found that approximately 75% of deaths were related to TB [12].

### Statistical Analysis

All adult TB cases were categorized as younger adults (ie, aged 15–64 years old) and older adults (ie, aged 65 years and older). Older adults were further stratified into two age groups: aged 65–74 years old and aged 75 years and older. We summarized demographic characteristics, diagnostic evaluation, and treatment outcomes with frequency and proportion for categorical variables and median and interquartile range (IQR) for continuous variables. Categorical variables were compared using Fisher’s exact test or χ^2^ test, as appropriate, and continuous variables using Mann-Whitney *U* test. Missing values were excluded from the analysis. Statistical significance was defined as *P* < .05.

A multivariable logistic regression model was constructed to assess for risk factors for TB treatment noncompletion among adults aged 65 years and older. The model was constructed using data from individuals who were alive at diagnosis and had public health charts available for review. Variables eligible for inclusion in the multivariable model included variables in the bivariate analyses with *P* < .1, or variables known in the published literature to be associated with TB treatment noncompletion (ie, age 75 years and older, sex, acid-fast bacilli [AFB] smear positivity as a proxy for bacillary burden, and radiographic evidence of cavitation) [13–15]. Individuals with missing data were excluded. Model collinearity was assessed with variance inflation factors. All statistical analyses were conducted using R Core Team (2021), version 4.1.2.

### Ethical Review

Because this analysis of TB surveillance and other routinely collected public health data were conducted to enable ACPHD to monitor, assess and inform local TB public health interventions, no human subject review was required.

## RESULTS

### Demographic and Clinical Characteristics

During January 1, 2016—December 31, 2019, a total of 517 adult TB cases were reported in Alameda County. Of these adult TB cases, 172 (33.3%) occurred among adults aged 65 years and older. Of the 172 older adults, 169 (98.3%) public health case management charts were available for review. Three individuals aged 65 and older did not have accessible public health case management charts and were excluded from those analyses.

In general, older adults were significantly more likely than younger adults to be male (68.6% vs 56.2%, *P* = .01), Asian (89.0% vs 67.0%, *P* < .001), and non-U.S. born (98.3% vs 88.1%, *P* < .001) (Table 1). Approximately 60% of all older patients were from three countries: China, Philippines, and India. Approximately 50% of older adults were publicly insured through Medi-Cal or Medicare, and 1.2% resided in long-term care facilities. Older adults had a higher prevalence of diabetes compared to younger adults (32.6% vs 20.9%, *P* = .01). Comorbid human immunodeficiency virus (HIV) infection was rare among older adults (0.6%); however, this age group was significantly more likely than younger adults to not be offered HIV testing (11.6% vs 3.8%, *P* < .001) or to have unknown results (4.7% vs 1.7%, *P* < .001). Among the older adult TB cases, CCI was higher in adults aged 75 years and older compared to adults aged 65–74 years old (median = 2 [IQR = 1–3] vs 1 [IQR = 0–2], *P* = .003), and the total number of non-TB medications were also higher among adults aged 75 years and older (median = 5 [IQR = 3–8] vs 3 [IQR = 1–6], *P* < .001).

**Table 1.** Demographic and Clinical Characteristics Among all Adult Tuberculosis Cases, by Age Group: Alameda County, 2016–2019

		All Adults			Older Adults		
Characteristics	Overall (*n* = 517)	15–64 (*n* = 345)	≥65 (*n* = 172)	*P* Value	65–74 (*n* = 71)	≥75 (*n* = 101)	*P* Value
Birth sex, *n* (%)							
Male	312 (60.3)	194 (56.2)	118 (68.6)	.01	48 (67.6)	70 (69.3)	.87
Race and Hispanic origin, *n* (%)				<.001			.94
Hispanic	65 (12.6)	57 (16.5)	8 (4.7)		4 (5.6)	4 (4.0)	
Asian	384 (74.3)	231 (67.0)	153 (89.0)		63 (88.7)	90 (89.1)	
Black non-Hispanic	36 (7.0)	31 (9.0)	5 (2.9)		2 (2.8)	3 (3.0)	
Native Hawaiian/Pacific Islander	8 (1.5)	8 (2.3)	0 (0.0)		0 (0.0)	0 (0.0)	
White non-Hispanic	21 (4.1)	15 (4.3)	6 (3.5)		2 (2.8)	4 (4.0)	
Multiple Race	2 (0.4)	2 (0.6)	0 (0.0)		0 (0.0)	0 (0.0)	
Non-US-born, *n* (%)	464 (89.7)	304 (88.1)	160 (98.3)	<.001	71 (100.0)	98 (97.0)	.14
Country of Birth, *n* (%)				<.001			.06
China	73 (14.1)	27 (7.8)	46 (26.7)		14 (19.7)	32 (31.7)	
Philippines	96 (18.6)	61 (17.7)	35 (20.3)		18 (25.4)	17 (16.8)	
India	92 (17.8)	69 (20.0)	23 (13.4)		13 (18.3)	10 (9.9)	
Vietnam	43 (8.3)	26 (7.5)	17 (9.9)		7 (9.9)	10 (9.9)	
Mexico	35 (6.8)	30 (8.7)	5 (2.9)		4 (5.6)	1 (1.0)	
Other	178 (34.4)	132 (38.3)	46 (26.7)		15 (21.1)	31 (30.7)	
Years in US before TB diagnosis, median (IQR)^a^	16.0 (4.5– 28.9)	11.5 (2.7–23.6)	24.0 (13.1–36.4)	<.001	19.2 (7.9–29.8)	27.7 (16.7–38.2)	<.001
Long-term care facility residence, *n* (%)^b^	3 (0.6)	1 (0.3)	2 (1.2)	.26	0 (0.0)	2 (2.0)	.51
Correctional facility residence, *n* (%)^b^	5 (1.0)	5 (1.4)	0 (0.0)	.18	…	…	…
Homeless, *n* (%)^b^	8 (1.6)	8 (2.3)	0 (0.0)	.06	…	…	…
Medi-Cal or Medicare insured, *n* (%)^c^	…	…	82 (48.5)	…	32 (45.1)	50 (51.0)	.15
Previous TB diagnosis, *n* (%)	46 (8.9)	25 (7.2)	22 (12.8)	.05	7 (9.9)	15 (14.9)	.37
Comorbid diabetes mellitus, *n* (%)	128 (24.8)	72 (20.9)	56 (32.6)	.01	25 (35.2)	31 (30.7)	.62
Comorbid dementia, *n* (%)^c^	…	…	15 (8.9)	…	3 (4.2)	12 (12.2)	.10
Comorbid hepatitis B, *n* (%)^c^	…	…	10 (5.9)	…	6 (8.5)	4 (4.1)	.31
Charlson comorbidity index, median (IQR)^c^	…	…	1 (0–3)	…	1 (0–2)	2 (1–3)	.003
Total non-TB medications, median (IQR)^c^	…	…	4 (2–7)	…	3 (1–6)	5 (3–8)	<.001
Site of Disease, *n* (%)				<.001			.68
Pulmonary	313 (60.5)	189 (55.1)	124 (72.1)		53 (74.6)	71 (70.3)	
Extrapulmonary	112 (21.7)	86 (25.1)	26 (15.1)		11 (15.5)	15 (14.9)	
Pulmonary and extrapulmonary	90 (17.4)	68 (19.8)	22 (12.8)		7 99.9)	15 (14.9)	

Abbreviations: IQR, interquartile range; TB, tuberculosis; US, United States.

a Denominator restricted to non-US-born cases only (*n* = 464).

b Residence at time of TB diagnosis.

c Variable collected from health department chart review for older adults only (*n* = 169); information was not abstracted for cases aged 15–64 years old.

### Comparison of Diagnostic Testing by Age Group

At the time of TB diagnosis, older adults were more likely than younger adults to have a negative interferon gamma release assay (IGRA) result (24.6% vs 16.0%, *P* = .01), and individuals aged 75 years and older had a higher proportion of negative IGRA results (32.9%) than those aged 65–74 years old (11.4%, *P* = .03) (Table 2). Of the older adults (*n* = 28) who had negative IGRA results, 21 (75%) were diagnosed through microbiological detection of *M tuberculosis* complex by a polymerase chain reaction assay or culture. Older adults were less likely to have any extrapulmonary involvement compared to younger adults (27.9% vs 44.9%, *P* < .001). Most older adults with pulmonary TB had AFB sputum smear-negative TB disease (58.0%), and 19.9% were culture- and nucleic acid amplification test (NAAT) assay-negative. Although sputum AFB culture was conducted for the vast majority of older adults (96.6%), 40.4% of this age group did not have a NAAT completed. Older adults, compared to younger adults, were less likely to have cavitary disease (18.6% vs 26.7%, *P* < .001) and more likely to have drug-susceptible TB disease (93.4% vs 81.3%, *P* = .001). No statistically significant differences in prevalence of isoniazid- or pyrazinamide (PZA)-resistant disease was detected among younger and older adults.

**Table 2.** Laboratory and Radiographic Characteristics Among All Adult Tuberculosis Cases, by Age Group: Alameda County, 2016–2019

		All Adults			Older Adults		
Characteristics	Overall (*n* = 517)	15–64 (*n* = 345)	≥65 (*n* = 172)	*P* Value	65–74 (*n* = 71)	≥75 (*n* = 101)	*P* Value
TST, *n* (%)							
Not done	439 (84.9)	283 (84.2)	156 (93.4)	.003	68 (95.8)	88 (91.7)	.36
TST-positive	53 (82.8)	45 (84.9)	8 (72.7)	.39	3 (100.0)	5 (62.5)	.49
IGRA, *n* (%)							
Not done	135 (26.1)	79 (23.1)	56 (32.9)	.03	27 (38.0)	29 (29.3)	.25
IGRA Result				.01			.03
Positive	285 (75.6)	210 (79.8)	75 (65.8)		34 (77.3)	41 (58.6)	
Negative	70 (18.6)	42 (16.0)	28 (24.6)		5 (11.4)	23 (32.9)	
Indeterminate	22 (5.8)	11 (4.2)	11 (9.6)		5 (11.4)	6 (8.6)	
Pulmonary AFB Sputum Smear, *n* (%)^a^							
Not done	7 (1.7)	4 (1.6)	3 (2.1)	.71	2 (3.3)	1 (1.2)	.57
Smear positive	168 (42.6)	108 (43.0)	60 (42.0)	.92	20 (34.5)	40 (47.1)	.17
Pulmonary AFB Sputum Culture, *n* (%)^a^							
Not done	10 (2.5)	5 (1.9)	5 (3.4)	.51	3 (5.0)	2 (2.3)	.65
Culture positive for TB	299 (74.2)	187 (71.1)	112 (80.0)	.26	42 (73.7)	70 (84.3)	.14
Extrapulmonary Tissue/Fluid Smear, *n* (%)^b^							
Not done	18 (8.9)	14 (9.1)	4 (8.3)	1.00	2 (11.1)	2 (6.7)	.62
Smear positive for TB	56 (30.4)	43 (30.7)	13 (29.5)	1.00	4 (25.0)	9 (32.1)	.74
Extrapulmonary Tissue/Fluid Culture, *n* (%)^b^							
Not done	29 (14.4)	21 (13.6)	8 (16.7)	.64	3 (16.7)	5 (16.7)	1.00
Culture positive for TB	124 (71.7)	91 (68.4)	33 (82.5)	.11	12 (80.0)	21 (84.0)	1.00
Nucleic Acid Amplification Testing, *n* (%)^a^							
Not done	144 (35.7)	85 (33.1)	59 (40.4)	.16	21 (35.0)	38 (44.2)	.31
Positive	197 (76.1)	123 (71.5)	74 (85.1)	.02	29 (74.4)	45 (93.8)	.02
Isoniazid-monoresistant, *n* (%)^c^	45 (10.8)	36 (13.5)	9 (6.0)	.02	5 (8.8)	4 (4.3)	.30
Pyrazinamide-monoresistant, *n* (%)^c^	11 (2.6)	10 (3.7)	1 (0.7)	.08	0 (0.0)	1 (1.1)	1.00
Rifampin-monoresistant, *n* (%)^c^	1 (0.2)	1 (0.4)	0 (0.0)	.05	0 (0.0)	0 (0.0)	…
Multidrug-resistant, *n* (%)^d^	6 (1.4)	6 (2.2)	0 (0.0)	.09	…	…	…
Abnormal imaging consistent with TB, *n* (%)^e^	466 (90.7)	299 (87.2)	167 (97.7)	.27	68 (95.8)	99 (99.0)	.41
Severe Disease Features, *n* (%)							
Radiographic findings of cavitary disease	124 (24.0)	92 (26.7)	32 (18.6)	<.001	16 (22.5)	16 (15.8)	.41
Radiographic findings of miliary disease	15 (2.9)	12 (3.5)	3 (1.7)	.18	2 (2.8)	1 (1.0)	.58

Abbreviations: AFB, acid-fast bacilli; IGRA, interferon-gamma release assay; TB, tuberculosis; TST, tuberculin skin test.

a Denominator restricted to cases with pulmonary involvement (*n* = 403).

b Denominator restricted to cases with extrapulmonary involvement (*n* = 202).

c Denominator restricted to cases with positive TB culture results on pulmonary or extrapulmonary specimen (*n* = 418).

d Denominator restricted to cases with positive TB culture results on pulmonary or extrapulmonary specimen (*n* = 418); multidrug resistance is defined as resistance to isoniazid and rifampin.

e Chest radiography or chest computed tomography scan.

### Treatment Outcomes by Age Group

No significant differences in initial TB regimen were found when comparing younger and older adult TB cases (Tables 3 and 4). One third (32.5%) of older adult TB cases experienced at least 1 adverse event, with similar proportions among cases aged 65–74 years old compared to aged 75 years and older (Table 3). The most frequently reported adverse events among older adult TB cases were elevated liver enzymes (56.6%), rash (32.1%), and nausea/vomiting (18.9%). Almost all older adult TB cases (96.4%) were initiated on PZA-containing regimens, a proportion that was similar among adults aged 65–74 years old and aged 75 years and older (Table 4). One third of older adult TB cases (32.1%) experienced at least one adverse event while on PZA, with no differences detected between older adult age groups. Of the older adult TB cases who had adverse events, 30.8% were rechallenged with PZA; half of whom subsequently tolerated eight weeks of PZA therapy. Older adults were also more likely to be dead at diagnosis than younger adults (3.5% vs 0.0%, *P* = .001); all six individuals who were diagnosed with TB postmortem were aged 65 years and older. Older adult TB cases, compared to younger adults, were significantly less likely to complete TB treatment (77.7% vs 88.4%, *P* = .001). Treatment completion was significantly lower among adult TB cases aged 75 years and older compared to adults aged 65–74 years old (72.9% vs 84.3% in those 65–74, *P* = .04) (Table 4). The proportion of deaths during TB treatment was significantly higher among older adults compared to younger adults (16.3% vs 2.9%, *P* < .001); adults aged 75 years and older, compared to aged 65–74 years old, were also more significantly likely to die during TB treatment (22.9% vs 7.1%, *P* = .01). When excluding all individuals who died during TB treatment, the proportion of younger and older adults who completed treatment was high (98.7% vs 96.6%) and similar (*P* = .24). When excluding only those who died in the first 8 weeks of treatment, we found similar results (91.1% in older adults vs 94.5% of younger adults failed to complete treatment, *P* = .18).

**Table 3.** Treatment and Adverse Events From Public Health Department Chart Review of Tuberculosis Cases Aged 65 Years and Older: Alameda County, 2016–2019

		Age Subgroups		
Characteristics	≥65 (*n* = 169)	65–74 (*n* = 71)	≥75 (*n* = 98)	*P* Value
Treatment duration in days, median (IQR)	202 (187–280)	202 (187–281)	202 (188–279)	.83
Culture conversion time in days, median (IQR)^a^	54 (33–63)	58 (39–66)	48 (42–62)	.25
Adverse event count, *n* (%)^a^				
At least one	53 (32.5)	25 (35.7)	28 (29.1)	.50
Two or more	10 (6.1)	3 (4.3)	7 (7.3)	.52
Adverse event type, *n* (%)^b^				
Rash	17 (32.1)	7 (28.0)	10 (35.7)	.77
Elevated liver enzymes	30 (56.6)	14 (56.0)	16 (57.1)	1.00
Nausea/vomiting/GI symptoms	10 (18.9)	1 (4.0)	9 (32.1)	.02
Hematologic abnormalities	6 (11.3)	2 (8.0)	4 (14.3)	.68
Drug-drug interactions	3 (5.7)	0 (0.0)	3 (10.7)	.24
PZA before first adverse event, *n* (%)^b^	52 (98.1)	25 (100.0)	27 (96.4)	1.00
PZA continued after first adverse event	16 (30.8)	7 (28.0)	9 (33.3)	.77
Completed ≥8 weeks of PZA after first adverse event	8 (50.0)	5 (71.4)	3 (33.3)	.32
PZA before second adverse event, *n* (%)^c^	1 (10.0)	0 (0.0)	1 (14.3)	1.00
PZA continued after second adverse event	1 (10.0)	0 (0.0)	1 (14.3)	1.00
Completed ≥8 weeks of PZA after second adverse event	1 (100.0)	…	1 (100.0)	…
RIPE regimen before first adverse event, *n* (%)^b^	48 (90.6)	25 (100.0)	23 (82.1)	.05
RIPE continued after first adverse event	7 (13.2)	2 (8.0)	5 (17.9)	.43

Abbreviations: GI, gastrointestinal; IQR, interquartile range; PZA, pyrazinamide; RIPE, rifampin, isoniazid, pyrazinamide, ethambutol.

a Denominator restricted to cases who were alive for treatment initiation (*n* = 163).

b Denominator restricted to cases experiencing at least one adverse event (*n* = 53).

c Denominator restricted to cases experiencing 2 or more adverse events (*n* = 10).

**Table 4.** Treatment and Outcome Characteristics Among All Adult Tuberculosis Cases, by Age Group: Alameda County, 2016–2019

		All Adults			Older Adults		
Characteristics	Overall (*n* = 517)	15–64 (*n* = 345)	≥65 (*n* = 172)	*P* Value	65–74 (*n* = 71)	≥75 (*n* = 101)	*P* Value
Directly observed TB therapy, *n* (%)^a^	297 (58.1)	199 (57.7)	98 (59.0)	.21	37 (52.9)	61 (63.5)	.44
Initiated on PZA-Containing Regimen, *n* (%)^a^	490 (95.8)	330 (95.7)	160 (96.4)	.20	68 (97.1)	92 (95.8)	.36
Completed ≥8 weeks of PZA Therapy, *n* (%)^b^	…	…	110 (68.8)	…	51 (75.0)	59 (64.1)	.17
At least one adverse event on PZA, *n* (%)^b^	…	…	52 (32.1)	…	25 (35.7)	27 (29.3)	.49
Completed TB therapy, *n* (%)^a^	434 (84.9)	305 (88.4)	129 (77.7)	.002	59 (84.3)	70 (72.9)	.04
Death during TB treatment, *n* (%)^a^	37 (2.0)	10 (2.9)	27 (16.3)	<.001	5 (7.1)	22 (22.9)	.01
Death at time of TB diagnosis, *n* (%)	6 (1.2)	0 (0.0)	6 (3.5)	.001	1 (1.4)	5 (5.0)	.40

Abbreviations: PZA, pyrazinamide; TB, tuberculosis.

a Denominator restricted to cases who were alive at treatment initiation (*n* = 511).

b Variable collected from health department chart review.

### Multivariable Model

The multivariable model was constructed using data from individuals who were aged 65 years and older, were alive at diagnosis, and had public health chart data available (*n* = 163). On bivariate analysis, aged 75 years and older, dementia, CCI, and positive AFB sputum smear at baseline were associated with increased odds of treatment noncompletion (Table 5). Initiation on a PZA-containing regimen was protective against treatment noncompletion in bivariate analyses (odds ratio [OR] = 0.18; 95% confidence interval [CI], .02–1.11; *P* = .06). In the adjusted model, dementia was significantly associated with treatment noncompletion (adjusted OR = 5.05; 95% CI, 1.33–20.32; *P* = .02). Collinearity between the variables in the multivariable model was insignificant, with all variance inflation factors <1.20.

**Table 5.** Factors Associated With Failure to Complete Treatment Among Tuberculosis Cases Aged 65 Years and Older: Alameda County, 2016–2019

Characteristics	OR	95% CI	*P* Value	aOR^a^	95% CI	*P* Value
Aged ≥75 years vs 65–74 years old	2.00	.93–4.56	.09	1.16	.45–3.07	.76
Male vs female	1.30	.59–3.06	.53	1.26	.47–3.65	.65
CCI, per 1 unit increase	1.29	1.08–1.55	.006	1.17	.94–1.46	.16
Dementia	4.86	1.62–14.96	.005	5.05	1.33–20.32	.02
AFB smear positive at baseline^b^	2.24	1.06–4.86	.07	2.06	.78–5.70	.15
Presence of cavity on chest radiography^c^	1.17	.44–2.92	.75	1.20	.39–3.50	.74
Initial PZA-containing regimen	0.18	.02–1.11	.06	1.01	.10–11.55	.99

Abbreviations: AFB, acid-fast bacilli; aOR, adjusted odds ratio; CCI, Charlson comorbidity index; CI, confidence interval; OR, odds ratio; PZA, pyrazinamide.

a Adjusted odds ratio calculated from a multivariable model containing all variables listed above.

b Sputum smear or other tissue sample smear.

c Chest x-ray or computed tomography scan.

## DISCUSSION

In a large county in Northern California, this study identified several clinical and public health challenges among older adults with TB. Advanced age was found to be associated with increased clinical complexity, negative diagnostic testing, and higher likelihood of poor outcomes including treatment noncompletion and death. Comorbidities important for this age group, in particular dementia, were associated with treatment noncompletion. At the same time, there were gaps in care, such as a large proportion that were identified who did not receive NAAT or HIV testing. Given that TB cases in the United States increasingly occur among older adults [16], these findings highlight the urgent need for greater evidence-based interventions to improve detection of and reduce TB-associated morbidity and mortality in this at-risk group.

Older adult TB cases in Alameda County differed demographically and clinically from their younger counterparts, exhibiting several risk factors for poor treatment outcomes as described in previous studies including older age, male sex, diabetes, and dementia [14]. Our older cohort was predominantly Asian, which has been associated with a higher prevalence of TB disease in the United States [16]. Older adults were also more likely to be non-US-born, potentially posing additional sociocultural challenges to TB care. Our findings of fewer older adults having received HIV and NAAT testing is consistent with prior literature [17] and raises concerns around the adequacy of TB screening and potential diagnostic delays. This delay may have contributed to a higher prevalence of postmortem TB diagnosis among older adults in our population, which has also been observed in previously published studies [18]. Other potential risk factors for higher proportion of posthumous TB diagnosis include nonspecific disease presentation [19] and false-negative IGRA testing in this age group associated with age-related anergy [20]. Previous literature has also reported higher proportions of AFB sputum smear-negative disease in older individuals due to paucibacillary disease [21], although our cohort did not exhibit differences in smear negativity between age groups. Smear negativity may be influenced by difficulties in obtaining specimens from individuals who are unable to expectorate or produce a sufficient sample, which may more often be the case for older adults. Efforts to increase diagnostic completeness in older TB cases are vital to making timely diagnoses and optimizing care.

Tuberculosis treatment regimens and duration were comparable between younger and older adults. Some clinicians have voiced concerns over using PZA-containing regimens in older adults because of potential adverse events, such as hepatotoxicity [22, 23]. However, many studies have shown that avoidance of PZA-containing regimens is associated with higher rates of treatment failure [24], higher mortality [12], and longer time to TB culture conversion [25]. Current US TB treatment guidelines recommend weighing treatment options on a case-by-case basis, taking into consideration individual disease characteristics, comorbidities, and potential drug interactions [26]. Our findings support this recommendation, because age alone did not appear to be associated with a different prevalence of PZA-related adverse events. Among those older adults who had adverse events and were rechallenged with PZA, half were able to tolerate treatment for 8 weeks. This finding has important implications because individuals who tolerate an 8-week course of PZA can have an overall shorter treatment course compared to those on non-PZA regimens. In line with current US guidance, comorbidity burden and other complicating disease characteristics, rather than age, may be more valuable in guiding the choice of a TB regimen and predicting treatment outcomes.

Despite having similar TB regimens and fewer instances of severe clinical features such as cavitation, older adults experienced TB treatment noncompletion and death at higher proportions than younger adults. Death during TB treatment was the main contributor to differences in treatment completion by age group; although we were limited in attributing deaths specifically to TB disease, past national surveillance work has found that the majority of deaths before treatment completion were due to TB [12]. These disparities in treatment completion may be further related to diagnostic and clinical differences between these age groups. As discussed, older adult cases were more likely to have incomplete or negative IGRAs and AFB sputum smears, factors that have been shown to be associated with treatment delays [27]. Adults in the older age group also more often had complicating comorbidities that are associated with impaired treatment response and higher mortality [28, 29]. It is notable that our multivariable model revealed a 5-fold increased odds of treatment noncompletion associated with comorbid dementia, consistent with past work that found an association between dementia and death from pulmonary TB, independent of age [30]. Older adults with dementia may experience diagnostic challenges due to inability to expectorate [31] and cognitive deficits that make treatment adherence difficult [32]. This group requires additional support and resources to successfully complete treatment, such as in-person directly observed therapy, and would benefit from additional research around non-sputum diagnostic methods.

Given the difficulty in TB diagnosis and poor outcomes including death for older adults, our findings highlight the importance of prevention through latent TB infection (LTBI) screening and treatment in this group. The vast majority of TB cases among older adults arise from LTBI reactivation (>95%), and studies suggest that LTBI prevalence is even higher in older adults with poorly controlled chronic conditions [33]. However, LTBI screening can be challenging for older adults, and TST and IGRA sensitivity declines as age increases [20, 34]. Limited clinical guidance exists around decisions to screen for LTBI among older patients with poorer health, and, to our knowledge, no reliable tools exist to predict progression of LTBI to active TB disease in this age group. Improved diagnostics and guidelines for shared decision making around LTBI screening and treatment are needed for this patient population [5].

These analyses are subject to several limitations. First, our data were derived from TB cases in Alameda County where the racial and ethnic population distribution may differ from other regions. Tuberculosis-complicating conditions such as diabetes may also have a different prevalence in this county compared to other regions of California or the United States. However, Alameda County has a high non-US-born population and encompasses a diverse range of urban and rural settings, potentially increasing the generalizability of our findings to other settings where TB is relevant. Second, our 3-year study period provides a recent snapshot of TB disease, but, notably, before the coronavirus disease-2019 (COVID-19) pandemic. Although additional factors may need to be considered during this period, we were still able to capture key aspects of TB disease in older adults that would most likely be exacerbated during the COVID-19 pandemic [35]. Third, our analyses were restricted to data available in our surveillance system and public health charts. As a result, we may not have identified all comorbidities or non-TB medications in the medical chart. We also could not definitively determine whether delays in diagnoses were due to provider or patient factors, and, as mentioned, we could not definitively attribute deaths to TB disease.

## CONCLUSIONS

Unique challenges exist in ensuring timely TB diagnosis and effective treatment in older adults, who may have higher rates of false-negative testing, treatment-complicating comorbidities, and high rates of treatment failure and death. As the burden of TB disease in the United States shifts toward an older patient population, public health practitioners and clinicians will be faced with evolving challenges in TB prevention and control. A greater understanding of characteristics and patterns in TB cases aged 65 and older will help guide research and interventions to improve TB care in this population.

## References

[1] Centers for Disease Control and Prevention . Table 1. Tuberculosis cases, case rates per 100,000 population, deaths, and death rates per 100,000 population, and percent change: United States, 1953–2019. Available at: https://www.cdc.gov/tb/statistics/reports/2019/table1.htm. Accessed 12 November 2021.

[2] Vaisman A , Barry P, Flood J. Assessing complexity among patients with tuberculosis in California, 1993–2016. Open Forum Infect Dis 2020; 7:ofaa264. doi:10.1093/ofid/ofaa264 PMC7415303 32793763

[3] Reichler MR , Khan A, Sterling TR, et al Risk and timing of tuberculosis among close contacts of persons with infectious tuberculosis. J Infect Dis 2018; 218:1000–8. doi:10.1093/infdis/jiy265 29767733 PMC6534268

[4] France AM , Grant J, Kammerer JS, Navin TR. A field-validated approach using surveillance and genotyping data to estimate tuberculosis attributable to recent transmission in the United States. Am J Epidemiol 2015; 182:799–807. doi:10.1093/aje/kwv121 26464470 PMC5996379

[5] Wu IL , Chitnis AS, Jaganath D. A narrative review of tuberculosis in the United States among persons aged 65 years and older. J Clin Tuberc Other Mycobact Dis 2022;28:100321. doi:10.1016/j.jctube.2022.100321 PMC9213239 35757390

[6] Hochberg NS , Horsburgh CR Jr. Prevention of tuberculosis in older adults in the United States: obstacles and opportunities. Clin Infect Dis 2013; 56:1240–7. doi:10.1093/cid/cit027 23362286 PMC3693488

[7] Kim S , Cohen T, Horsburgh CR, Miller JW, Hill AN, Marks SM, et al Trends, mechanisms, and racial/ethnic differences of tuberculosis incidence in the US-born population aged 50 years or older in the United States. Clin Infect Dis 2022; 74:1594–603. doi:10.1093/cid/ciab668 34323959 PMC8799750

[8] U.S. National Center for Health Statistics . Alameda County, California: 2020. 2020 Decennial Census. U.S. Census Bureau; 2022. Available at: https://data.census.gov/cedsci/profile? g=0500000US06001. Accessed 5 May 2022.

[9] Alameda County Public Health Department . Tuberculosis Fact Sheet – Alameda County, 2021. Available at https://acphd-web-media.s3-us-west-2.amazonaws.com/media/data-reports/communicable-disease/docs/tb-fact-sheet-alameda-2021.pdf. Accessed 5 May 2022.

[10] Centers for Disease Control and Prevention . CDC tuberculosis surveillance data training - Report of Verified Case of Tuberculosis. https://www.cdc.gov/tb/programs/rvct/InstructionManual.pdf. Accessed 1 November 2021.

[11] Charlson ME , Pompei P, Ales KL, MacKenzie CR. A new method of classifying prognostic comorbidity in longitudinal studies: development and validation. J Chronic Dis 1987; 40:373–83. doi:10.1016/0021-9681(87)90171-8 3558716

[12] Beavers SF , Pascopella L, Davidow AL, et al Tuberculosis mortality in the United States: epidemiology and prevention opportunities. Ann Am Thorac Soc 2018; 15:683–92. doi:10.1513/AnnalsATS.201705-405OC 29490150 PMC6531349

[13] Urbanowski ME , Ordonez AA, Ruiz-Bedoya CA, Jain SK, Bishai WR. Cavitary tuberculosis: the gateway of disease transmission. Lancet Infect Dis 2020; 20:e117–28. doi:10.1016/S1473-3099(20)30148-1 32482293 PMC7357333

[14] Yamana H , Matsui H, Fushimi K, Yasunaga H. Treatment options and outcomes of hospitalized tuberculosis patients: a nationwide study. Int J Tuberc Lung Dis 2015; 19:120–6. doi:10.5588/ijtld.14.0333 25519801

[15] Kim TC , Blackman RS, Heatwole KM, Kim T, Rochester DF. Acid-fast bacilli in sputum smears of patients with pulmonary tuberculosis. Prevalence and significance of negative smears pretreatment and positive smears post-treatment. Am Rev Respir Dis 1984; 129:264–8. 6421211

[16] Filardo TD , Feng P, Pratt RH, Price SF, Self JL. Tuberculosis United States, 2021. MMWR Morb Mortal Wkly Rep 2022; 71:441–6. doi:10.15585/mmwr.mm7112a1 35324877 PMC8956339

[17] Reddy D , Walker J, White LF, Brandeis GH, Russell ML, Horsburgh CR, et al Latent tuberculosis infection testing practices in long-term care facilities, Boston, Massachusetts. J Am Geriatr Soc 2017; 65:1145–51. doi:10.1111/jgs.14696 28467605

[18] Pratt RH , Winston CA, Kammerer JS, Armstrong LR. Tuberculosis in older adults in the United States, 1993–2008. J Am Geriatr Soc 2011; 59:851–7. doi:10.1111/j.1532-5415.2011.03369.x 21517786

[19] Abbara A , Collin SM, Kon OM, et al Time to diagnosis of tuberculosis is greater in older patients: a retrospective cohort review. ERJ Open Res 2019; 5:00228–2018. doi:10.1183/23120541.00228-2018 31720296 PMC6826249

[20] Yamasue M , Komiya K, Usagawa Y, et al Factors associated with false negative interferon-γ release assay results in patients with tuberculosis: a systematic review with meta-analysis. Sci Rep 2020; 10:1607. doi:10.1038/s41598-020-58459-9 32005930 PMC6994686

[21] Shah NS , Cavanaugh JS, Pratt R, Cain KP, Wells C, Laserson K, et al Epidemiology of smear-negative pulmonary tuberculosis in the United States, 1993–2008. Int J Tuberc Lung Dis 2012; 16:1234–40. doi:10.5588/ijtld.11.0794 22748057

[22] Cooper R , Houston S, Hughes C, Johnston JC. Chapter 10: Treatment of active tuberculosis in special populations. Can J Respir Crit Care Sleep Med 2022; 6(Suppl 1):149–66.

[23] Gardner Toren K , Spitters C, Pecha M, Bhattarai S, Horne DJ, Narita M. Tuberculosis in older adults: Seattle and King County, Washington. Clin Infect Dis 2020; 70:1202–7. doi:10.1093/cid/ciz306 30977788

[24] Richardson NL . Evaluating provider prescribing practices for the treatment of tuberculosis in Virginia, 1995 to 1998: an assessment of educational need. J Contin Educ Health Prof 2000; 20:146–55. doi:10.1002/chp.1340200303 11232250

[25] Hagiwara E , Suido Y, Asaoka M, et al Safety of pyrazinamide-including regimen in late elderly patients with pulmonary tuberculosis: a prospective randomized open-label study. J Infect Chemother 2019; 25:1026–30. doi:10.1016/j.jiac.2019.05.030 31229376

[26] Nahid P , Dorman SE, Alipanah N, et al Official American Thoracic Society/Centers for Disease Control and Prevention/Infectious Diseases Society Of America Clinical Practice Guidelines: Treatment of Drug-Susceptible Tuberculosis. Clin Infect Dis 2016; 63:e147–95. doi:10.1093/cid/ciw376 27516382 PMC6590850

[27] Nguyen DT , Teeter LD, Graves J, Graviss EA. Characteristics associated with negative interferon-γ release assay results in culture-confirmed tuberculosis patients, Texas, USA, 2013–2015. Emerg Infect Dis 2018; 24:534–40. doi:10.3201/eid2403.171633 29460756 PMC5823348

[28] Nguyen CH , Pascopella L, Barry PM. Association between diabetes mellitus and mortality among patients with tuberculosis in California, 2010–2014. Int J Tuberc Lung Dis 2018; 22:1269–76. doi:10.5588/ijtld.18.0011 30355405

[29] Kumar AKH , Chandrasekaran V, Kannan T, et al Anti-tuberculosis drug concentrations in tuberculosis patients with and without diabetes mellitus. Eur J Clin Pharmacol 2017; 73:65–70. doi:10.1007/s00228-016-2132-z 27651240

[30] Saiki M , Iijima Y, Honda T, et al Coexistence of dementia with smear-positive pulmonary tuberculosis is associated with patient in-hospital mortality. Respir Investig 2019; 57:354–60. doi:10.1016/j.resinv.2019.01.004 30760407

[31] Jackson DA , Mailer K, Porter KA, et al Challenges in assessing transmission of Mycobacterium tuberculosis in long-term-care facilities. Am J Infect Control 2015; 43:992–6. doi:10.1016/j.ajic.2015.03.035 25952618 PMC4635053

[32] Bea S , Lee H, Kim JH, et al Adherence and associated factors of treatment regimen in drug-susceptible tuberculosis patients. Front Pharmacol 2021; 12:625078. doi:10.3389/fphar.2021.625078 33790788 PMC8005597

[33] Huang HL , Huang WC, Lin KD, et al Completion rate and safety of programmatic screening and treatment for latent tuberculosis infection in elderly patients with poorly controlled diabetic mellitus: a prospective multicenter study. Clin Infect Dis 2021; 73:e1252–60. doi:10.1093/cid/ciab209 33677558 PMC8442788

[34] Almeida Santos J , Duarte R, Nunes C. Tuberculin skin test and predictive host factors for false-negative results in patients with pulmonary and extrapulmonary tuberculosis. Clin Respir J 2020; 14:541–8. doi:10.1111/crj.13166 32052551

[35] Nabity SA , Han E, Lowenthal P, et al Sociodemographic characteristics, comorbidities, and mortality among persons diagnosed with tuberculosis and COVID-19 in close succession in California, 2020. JAMA Netw Open 2021; 4:e2136853. doi:10.1001/jamanetworkopen.2021.36853 PMC8642782 34860244

